# Supplementary figures and images for: Structural diversity of B-cell receptor repertoires along the B-cell differentiation axis in humans and mice
Source: PLoS Comput Biol. 2020 Feb 18;16(2):e1007636. doi: 10.1371/journal.pcbi.1007636 (PMC7048297; doi:10.1371/journal.pcbi.1007636)

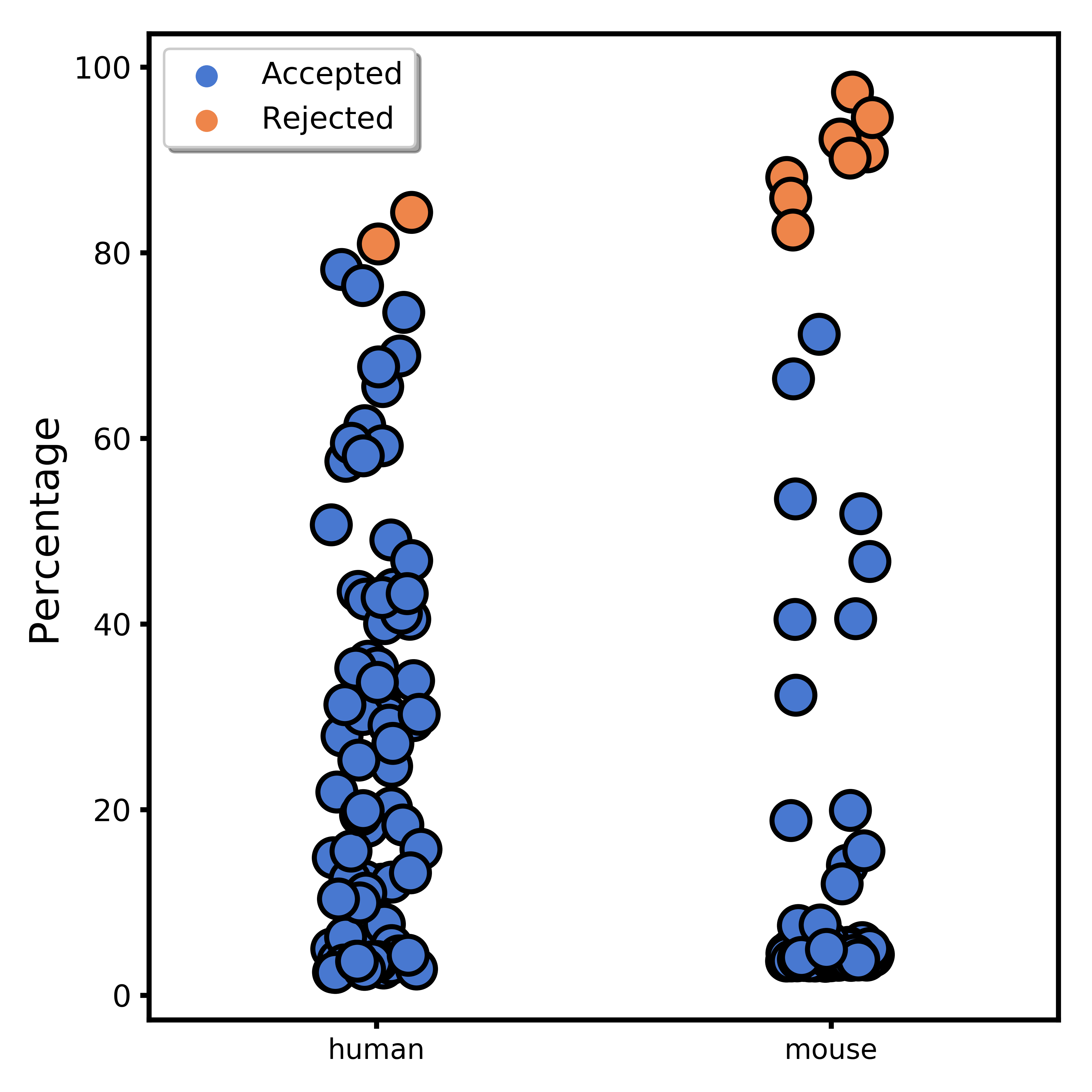

Supplement: S1 Fig — Each circle represents the percentage of sequences present in the two most redundant CDR-H3 clusters in a BCR repertoire. If this percentage exceeds 80% of the total number of that BCR repertoire’s sequences (orange circle), the BCR repertoire was not included in the subsequent structural analysis. Different thresholds were checked, lower percentage cut-offs produced the same qualitative results in our structural analysis. Hence, 80% cut-off was selected to retain as many BCR repertories as possible. (TIF) [file pcbi.1007636.s001.tif]

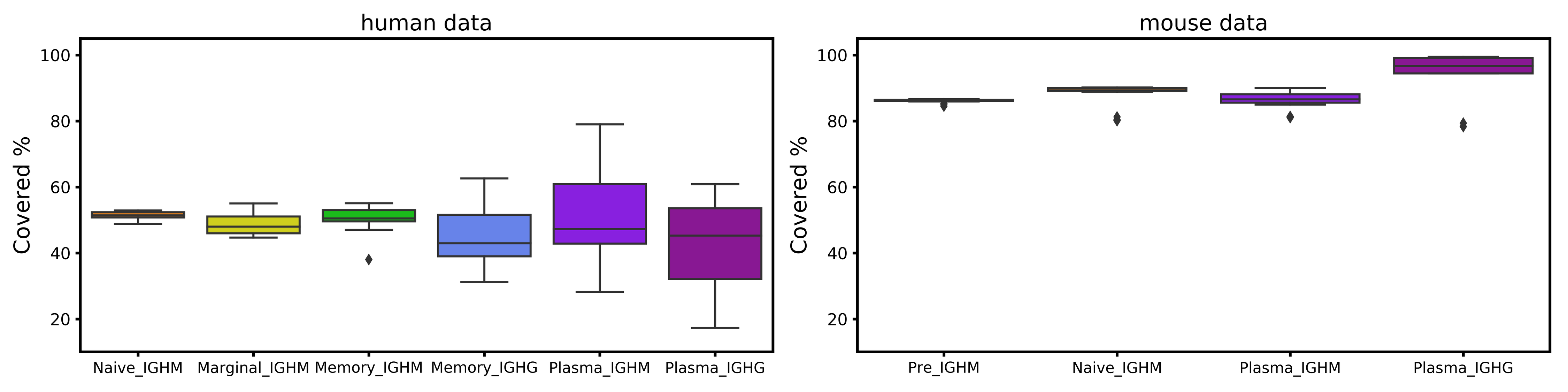

Supplement: S2 Fig — CDR-H3 structural coverage in human (A) and mouse (B) BCR repertoires. FREAD CDR-H3 modellability was calculated across BCR repertoires of different B-cell types. FREAD modellability is defined as the percentage of sequences with predicted CDR-H3 structures over the total number of sequences in a given BCR repertoire. (TIF) [file pcbi.1007636.s002.tif]

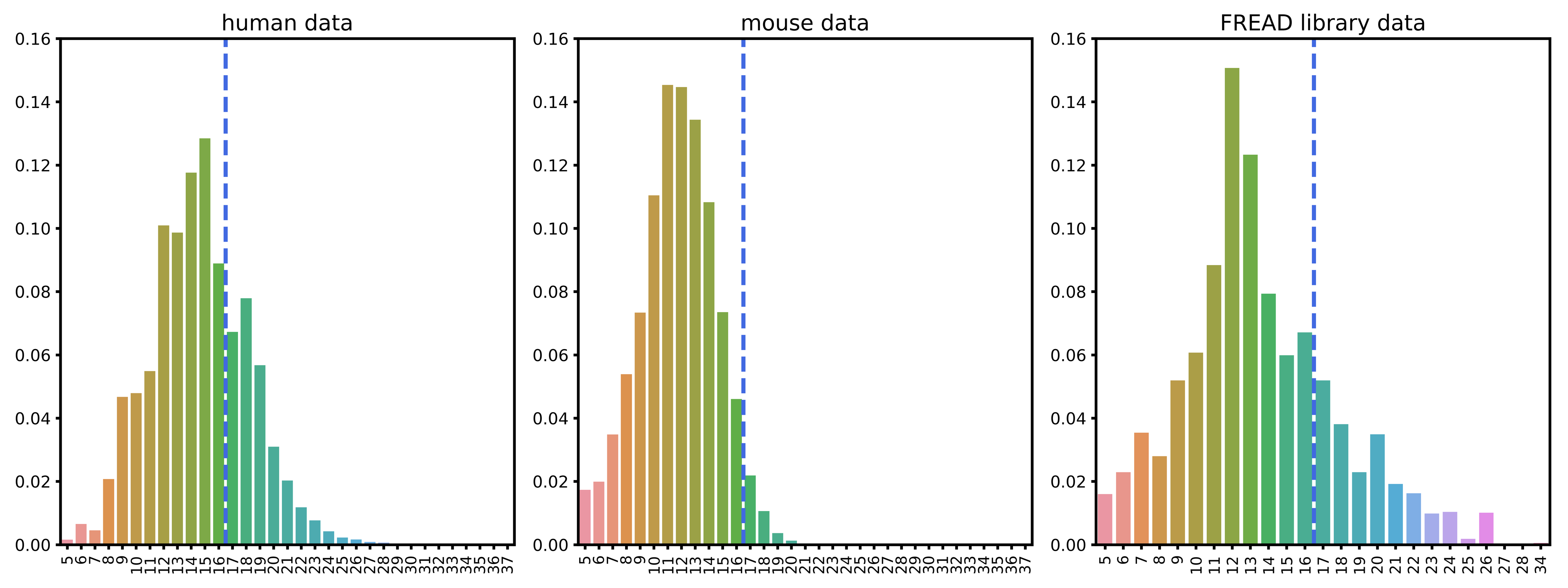

Supplement: S3 Fig — Normalized CDR-H3 length distribution was calculated for all BCR repertoires in the human and mouse data, and all CDR-H3 templates that were in our FREAD library. Mouse CDR-H3s were on average shorter than human. The distribution of FREAD CDR-H3 lengths is different that found in the human and mouse data. The vertical blue line shows our CDR-H3 length cut-off (17 residues). Sequences and FREAD templates whose CDR-H3 lengths were longer than the cut-off were not considered in our analysis. (TIF) [file pcbi.1007636.s003.tif]

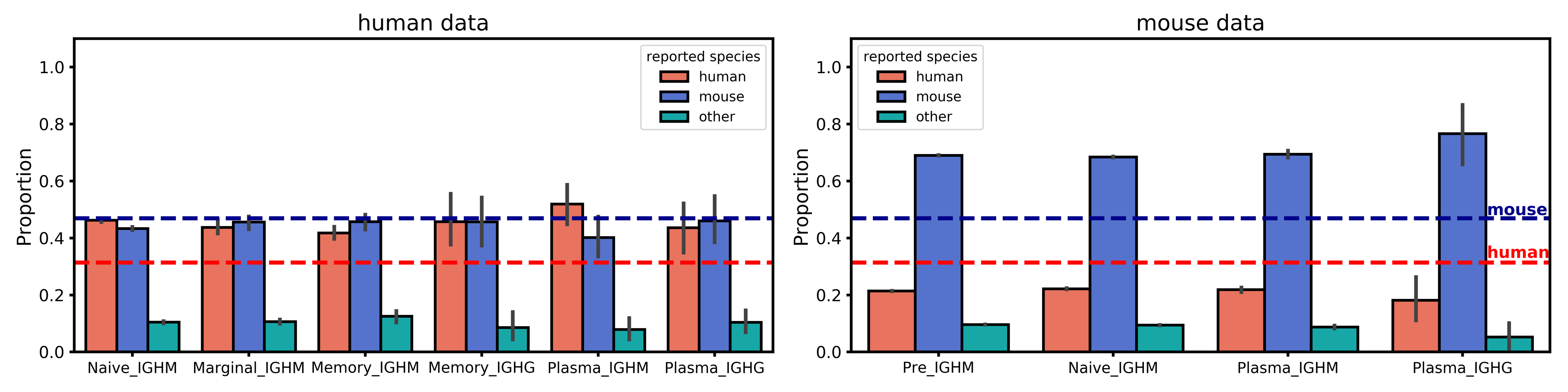

Supplement: S4 Fig — We calculated the proportion of CDR-H3 template reported species of origin for every BCR repertoire across different B-cell types. Reported species origin information was extracted from SAbDab (10). Orange bars show the proportion of human CDR-H3 templates, blue bars represent mouse CDR-H3 templates, while cyan bars depict the proportion of other than human or mouse CDR-H3 templates. The horizontal lines represent the expected outcome of uniform sampling of human (orange) and mouse (blue) CDR-H3 templates. Uniform sampling was calculated as the number of human or mouse CDR-H3 templates over the total number CDR-H3 templates found in our FREAD library. (TIF) [file pcbi.1007636.s004.tif]

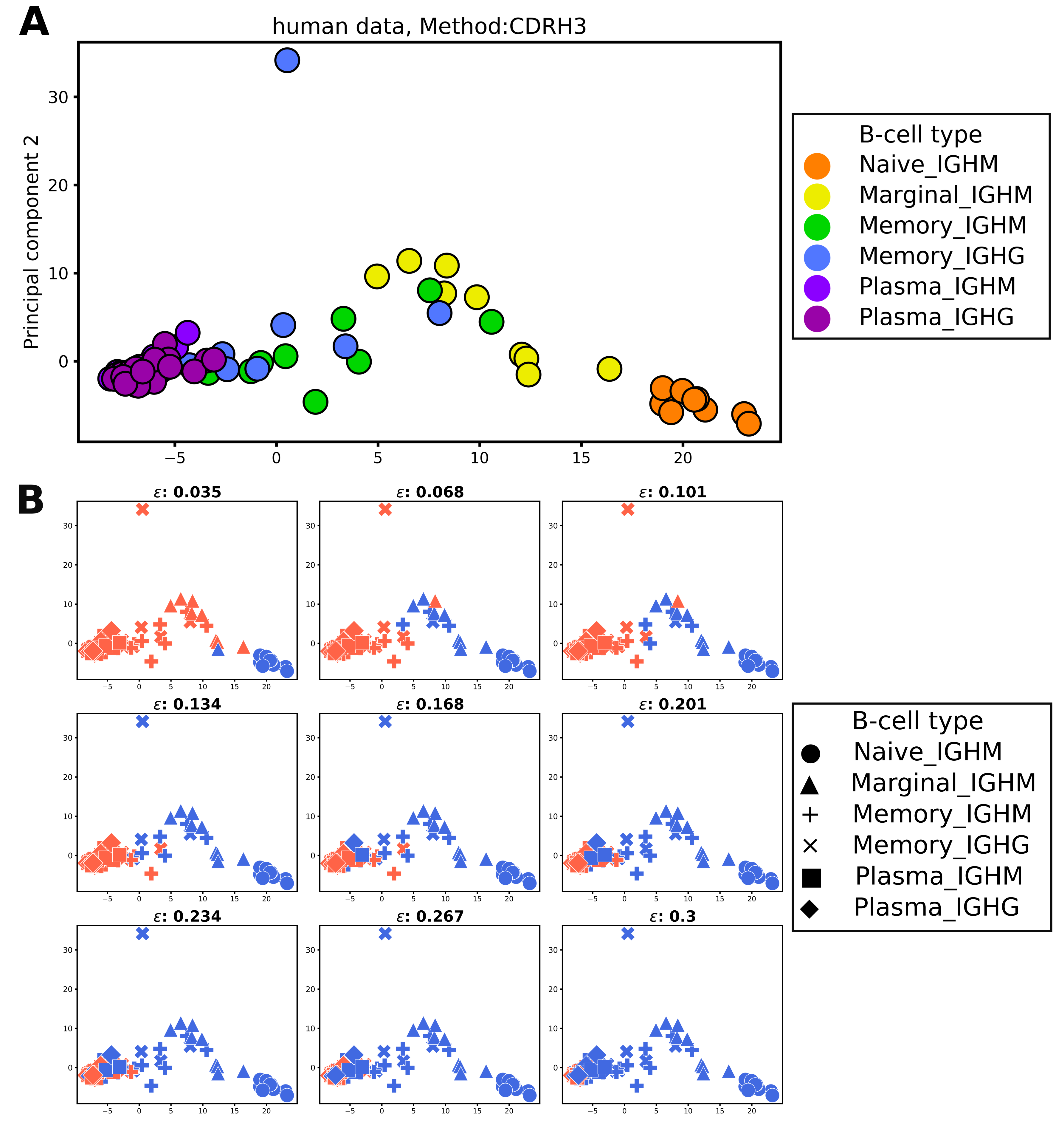

Supplement: S5 Fig — (a) PCA was performed on the human BCR repertoires (circles) with CDR-H3 cluster usages used as the features. The first two principal components were used to visualize any separation. Colours represent different B-cell types. (b) DBSCAN analysis with increasing maximum distance (ε) was employed to interrogate CDR-H3 cluster usage densities across human BCR repertoires. PCA analysis (as in a) was then used to visualize the DBSCAN clustering. The parameter ε was increased left-to-right, top-to-bottom. Marker shapes indicate different B-cell types; blue colour represents BCR repertoires that clustered with antigen-unexperienced BCR repertoires (naïve); orange colour shows DBSCAN-unclustered BCR repertoires at that ε value. (TIF) [file pcbi.1007636.s005.tif]

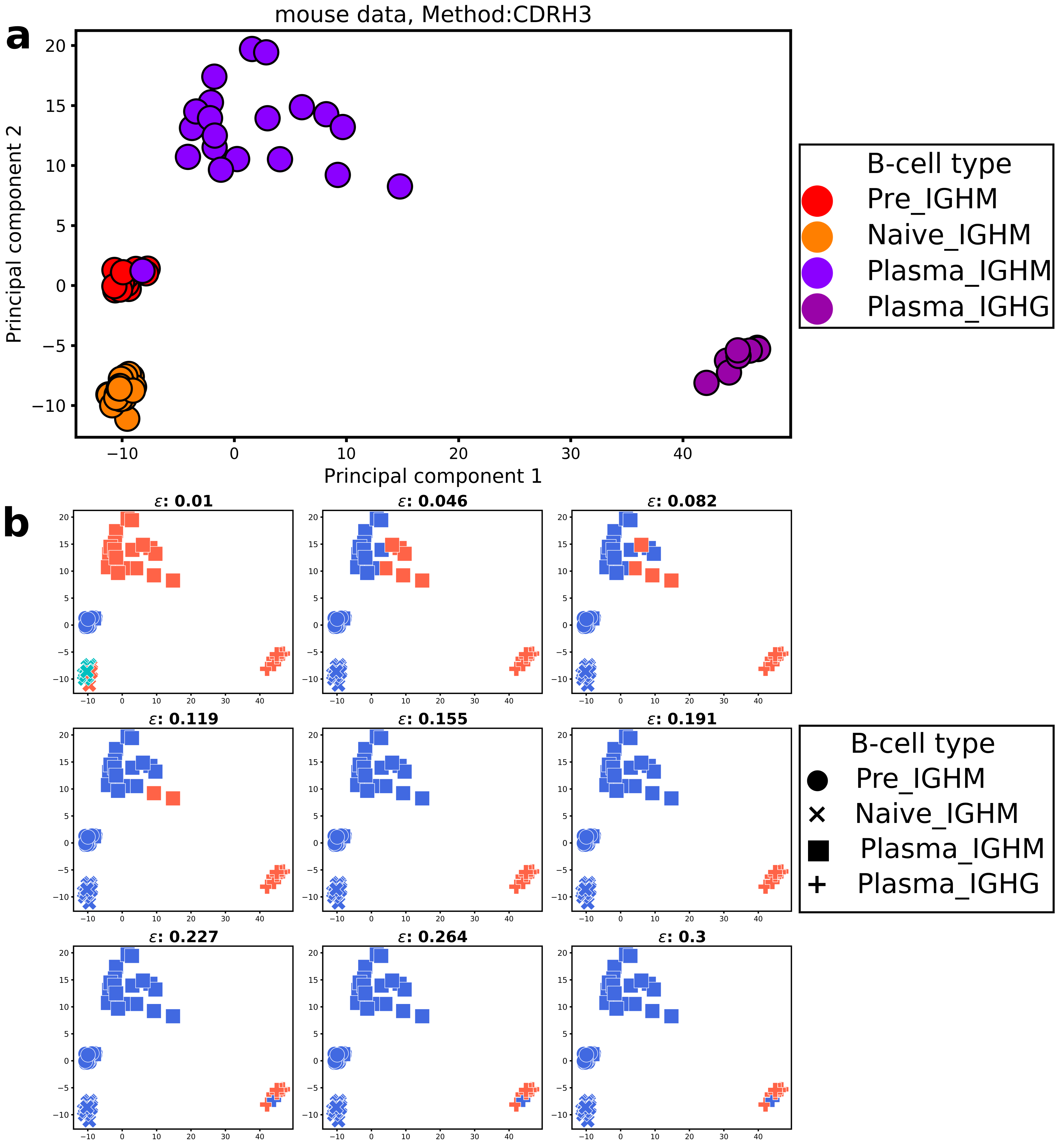

Supplement: S6 Fig — (a) PCA was performed on the mouse BCR repertoires (circles), with CDR-H3 cluster usages used as the features. The first two principal components were used to visualize any separation. Colours represent different B-cell types. (b) DBSCAN analysis with increasing maximum distance (ε) was employed to interrogate CDR-H3 usage densities across mouse BCR repertoires. PCA analysis (as in a) was then used to visualize the DBSCAN clustering. The parameter ε was increased left-to-right, top-to-bottom. Marker shapes indicate different B-cell types; cyan colour (in the top left subplot) represents naïve BCR repertoires, blue colour represents BCR repertoires that clustered with antigen-unexperienced BCR repertoires (pre and naïve); orange colour shows DBSCAN-unclustered BCR repertoires at that ε value. (TIF) [file pcbi.1007636.s006.tif]

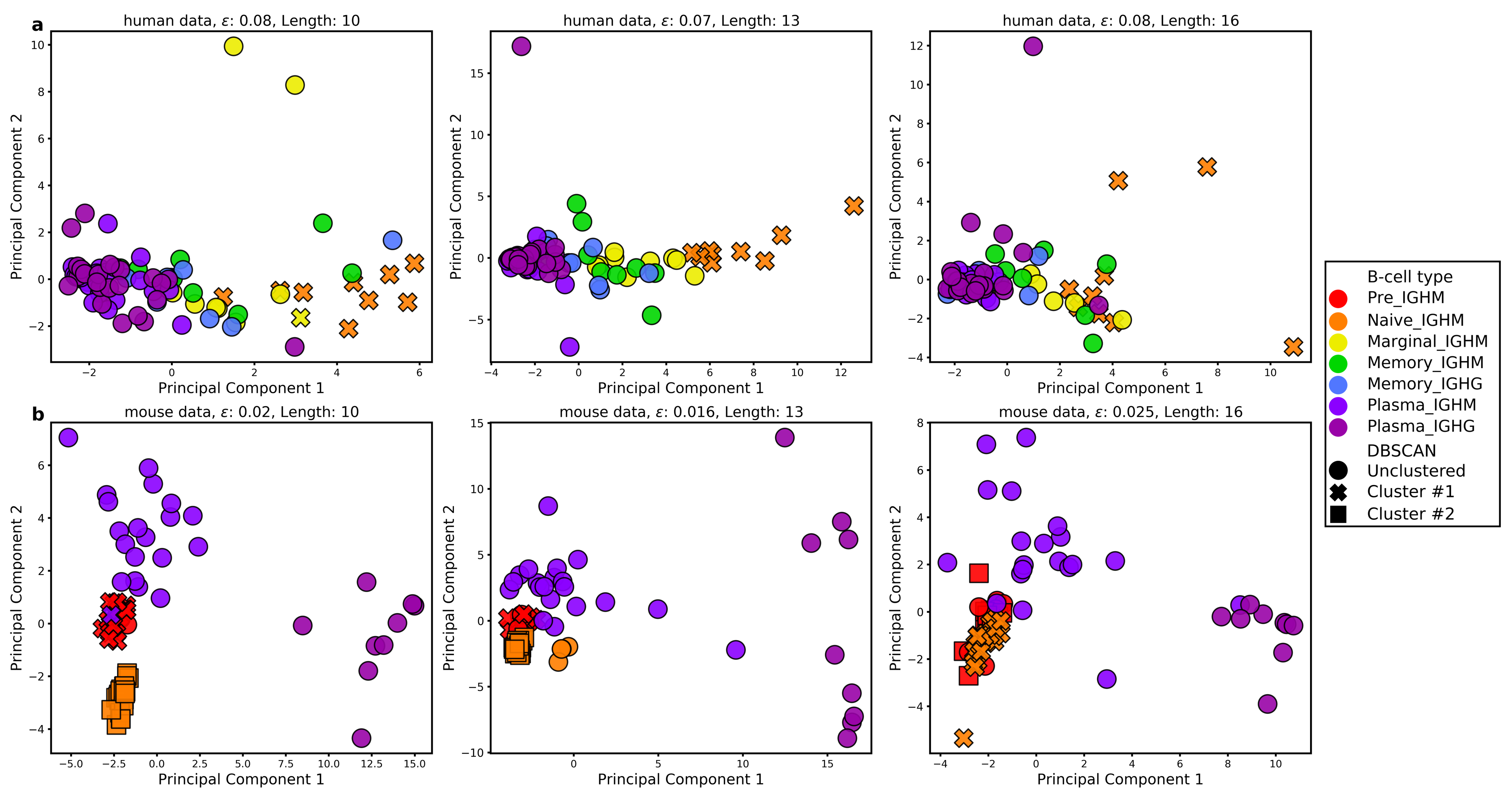

Supplement: S7 Fig — PCA was performed on (a) human and (b) mouse BCR repertoires, with CDR-H3 cluster usages selected as the features. The first two principal components were used to visualize any separation. Colours represent different B-cell types. DBSCAN was employed to quantify densities of CDR-H3 cluster usages across the repertoires. Marker shapes illustrate DBSCAN cluster information. Circle markers indicate DBSCAN unclustered BCR repertoires; other marker shapes show individual DBSCAN clusters. We considered a B-cell type separation if naïve and pre (antigen-unexperienced) BCR repertoires displayed the closest densities of CDR-H3 cluster usages at lower ε values in DBSCAN. In both (a) human and (b) mouse repertoires, antigen-unexperienced B-cell types cluster first regardless of CDR-H3 lengths. This confirms that BCR repertoires of different B-cell types have different patterns of CDR-H3 cluster usage. (TIF) [file pcbi.1007636.s007.tif]

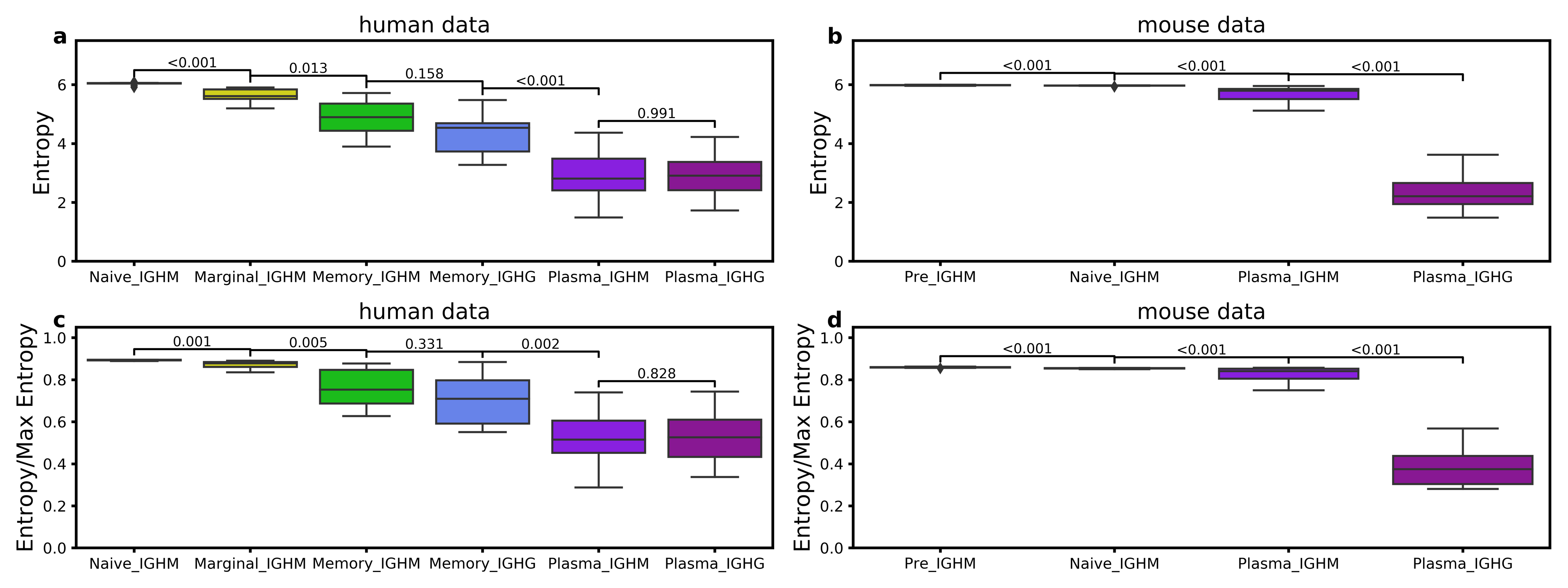

Supplement: S8 Fig — Shannon entropy was calculated for CDR-H3 cluster usage in human (a) and mouse (b) data. To account for the varying numbers of CDR-H3 clusters across B-cell types, structural diversity of CDR-H3s was expressed as a proportion of entropy over theoretical maximum entropy in the human (c) and mouse (d) data. Theoretical maximum entropy was found for each BCR repertoire by using CDR-H3 structures represented in the given repertoire in equal proportions in entropy calculations. Higher values indicate higher diversities of CDR-H3 cluster usage. The Mann-Whitney U-test was used for statistical analysis and p-values are reported. (TIF) [file pcbi.1007636.s008.tif]

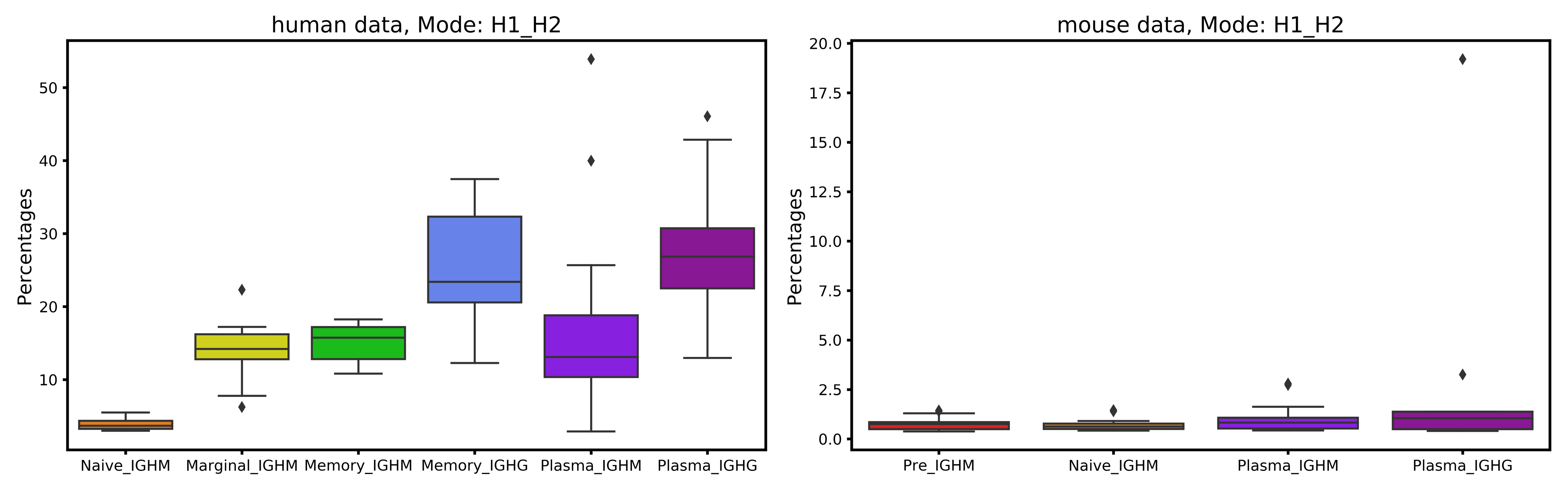

Supplement: S9 Fig — Canonical class divergence was defined as a mismatch in either the CDR-H1 or CDR-H2 canonical class from the germline canonical class. Percentages were calculated as the number of sequences with canonical class divergence over the total number of sequences in a given BCR repertoire. Colours represent different B-cell types. (TIF) [file pcbi.1007636.s009.tif]

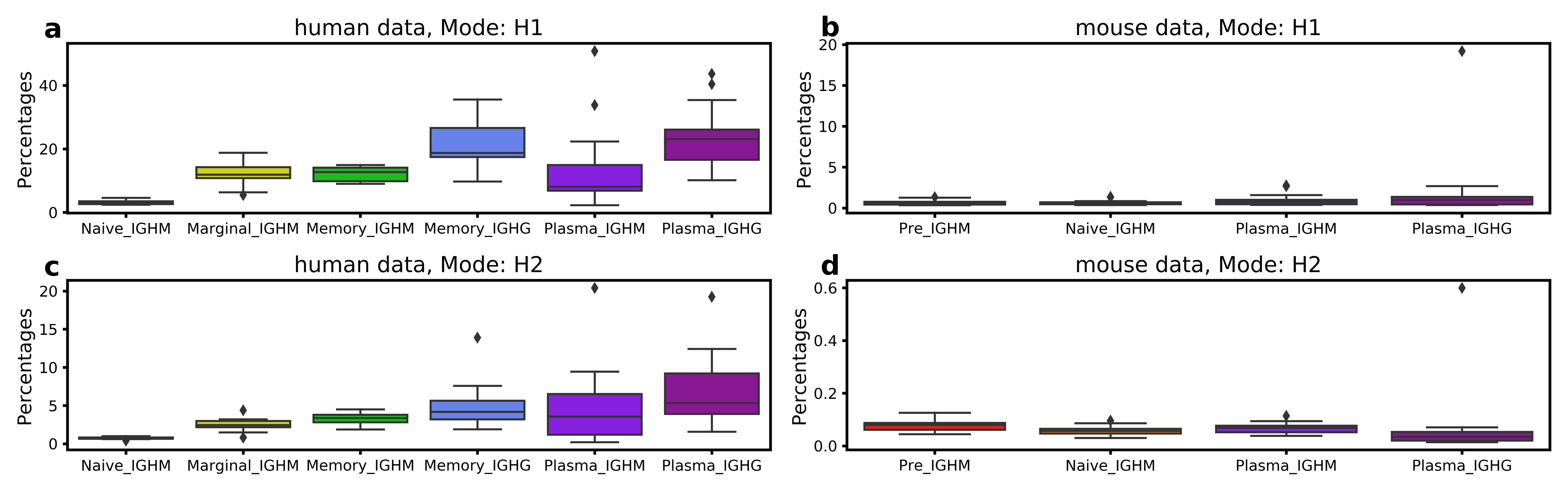

Supplement: S10 Fig — The top boxplots show canonical class divergence in CDR-H1 canonical class from the germline canonical class in the (a) human and (b) mouse data. Percentages were calculated as the number of sequences with canonical class divergence over the total number of sequences in a given BCR repertoire. The bottom boxplots show the same analysis on CDR-H2 canonical class in the (c) human and (d) mouse data. Colours represent different B-cell types. (TIF) [file pcbi.1007636.s010.tif]

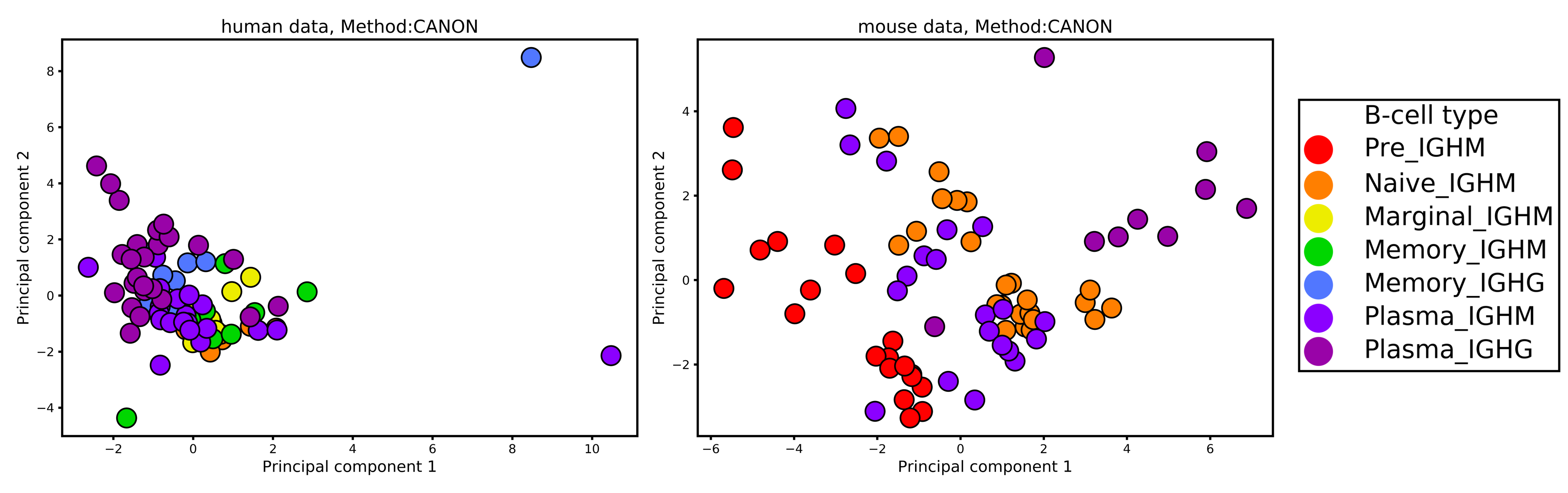

Supplement: S11 Fig — Features included in the PCA were frequencies of CDR-H1 and CDR-H2 combinations in BCR repertoires. The first two principal components were used to visualize the separation of BCR repertoires. Colours represent different B-cell types. (TIF) [file pcbi.1007636.s011.tif]

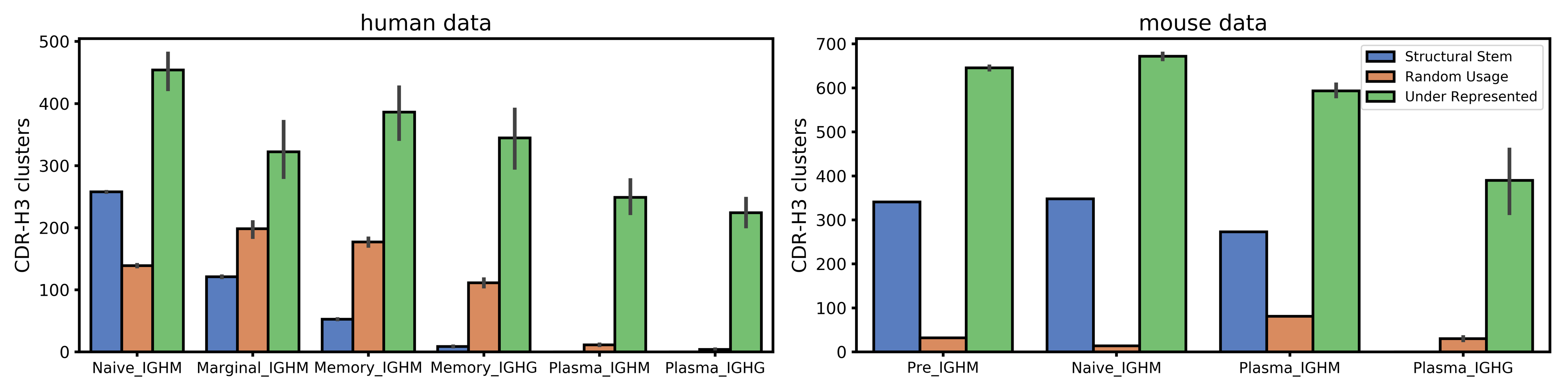

Supplement: S12 Fig — Structural Stems (blue bars) were defined as CDR-H3 clusters, which were over-represented across BCR repertoires of the same B-cell type. Under-Represented (green bars) were under-represented CDR-H3 clusters. CDR-H3 clusters, whose usages were not significantly different from random sampling, were termed Random-Usage (orange bars). The X-axis shows different B-cell types in the order of the B-cell maturation axis. The Y-axis shows the number of CDR-H3 clusters. (TIF) [file pcbi.1007636.s012.tif]

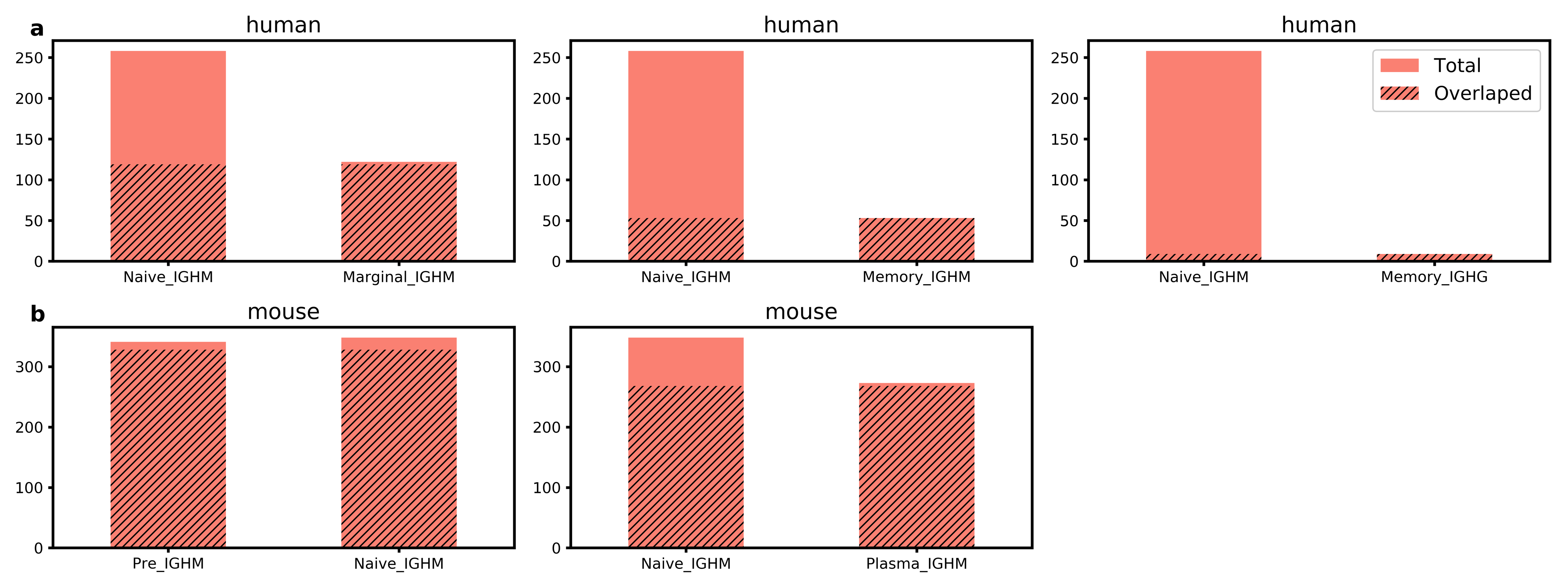

Supplement: S13 Fig — Naïve and antigen experienced BCR repertoires were investigated for the Structural Stem overlap in the human (row A) and mouse (row B) data. The overlap was defined as a number of shared clusters between Structural Stem CDR-H3 clusters in BCR repertoires found in two different B-cell types. The X-axis shows the B-cell types. The Y-axis shows the total number of Structural Stem CDR-H3 clusters. The stripped pattern indicates the number of overlapped Structural Stem CDR-H3 clusters. (TIF) [file pcbi.1007636.s013.tif]
