# Supplementary material for: Structural diversity of B-cell receptor repertoires along the B-cell differentiation axis in humans and mice
Source: PLoS Comput Biol. 2020 Feb 18;16(2):e1007636. doi: 10.1371/journal.pcbi.1007636 (PMC7048297; doi:10.1371/journal.pcbi.1007636)
Supplement: S1 Table — FREAD performance of CDR-H3 structure prediction was validated on the human and mouse data across three CDR-H3 length bins: 5 to 12, 13 and 14, and 15 and 16. For each length bin, ESS cut-offs were selected to achieve an average RMSD better than 3 Å or a coverage greater than 15%. The same ESS cut-offs were selected for both human and mouse data. Precision was defined as the percentage of FREAD predictions within 3 Å over the total number of predictions within the ESS cut-off. (DOCX) [file pcbi.1007636.s014.docx]

| Data |  |  |
| --- | --- | --- |
| Human | RMSD | 2.5 Å |
|  | Precision | 68.8% |
| Mouse | RMSD | 2.5 Å |
|  | Precision | 68.4% |

Supplementary Table 1. **Estimated FREAD average RMSD and precision on the human and mouse data**. FREAD performance of CDR-H3 structure prediction was validated on the human and mouse data across three CDR-H3 length bins: 5 to 12, 13 and 14, and 15 and 16. For each length bin, ESS cutoffs were selected to achieve an average RMSD better than 3 Å or a coverage greater than 15%. The same ESS cutoffs were selected for both human and mouse data. Precision was defined as the percentage of FREAD predictions within 3 Å over the total number of predictions within the ESS cutoff.
