# Supplementary material for: Structural diversity of B-cell receptor repertoires along the B-cell differentiation axis in humans and mice
Source: PLoS Comput Biol. 2020 Feb 18;16(2):e1007636. doi: 10.1371/journal.pcbi.1007636 (PMC7048297; doi:10.1371/journal.pcbi.1007636)
Supplement: S2 Table — Annotation was performed on the human and mouse data. The human data contained 5.7 million sequences with CDR-H3 loop lengths of 16 amino acids or shorter. SCALOP predicted CDR-H1 loop shapes in 97.7% of sequences and CDR-H2 loop shapes in 95.4% in the human data. The total number of mouse sequences was ~207 million, of which 99% of CDR-H1 and ~100% of CDR-H2 loop shapes were annotated. (DOCX) [file pcbi.1007636.s015.docx]

| Data | Total sequences | CDR-H1 annotated | CDR-H2 annotated |
| --- | --- | --- | --- |
| Human | 5,712,939 | 5,598,599 (97.7%) | 5,425,279 (95.4%) |
| Mouse | 206,680,496 | 204,805,604 (99%) | 206,592,576 (~100%) |

Supplementary Table 2. **SCALOP annotation of Ig-seq data**. Annotation was performed on the human and mouse data. The human data contained 5.7 million sequences with CDR-H3 loop lengths of 16 amino acids or shorter. SCALOP predicted CDR-H1 loop shapes in 97.7% of sequences and CDR-H2 loop shapes in 95.4% in the human data. The total number of mouse sequences was ~207 million, of which 99% of CDR-H1 and ~100% of CDR-H2 loop shapes were annotated.
