# Supplementary material for: Structural diversity of B-cell receptor repertoires along the B-cell differentiation axis in humans and mice
Source: PLoS Comput Biol. 2020 Feb 18;16(2):e1007636. doi: 10.1371/journal.pcbi.1007636 (PMC7048297; doi:10.1371/journal.pcbi.1007636)
Supplement: S1 Appendix — (DOCX) [file pcbi.1007636.s017.docx]

# S1 Appendix

## Species CDR-H3 template usage

To identify portions of structural space that were never seen in the human or mouse data, we searched for CDR-H3 clusters that were never utilized in the human and mouse data, recording all CDR-H3 templates belonging to these clusters. The number of such templates was 109 (~4% of all FREAD templates). Eighty-eight of the 109 unused CDR-H3 templates derived from nanobodies, which constituted ~32% of all nanobody CDR-H3 loops in our FREAD library. A further six unused templates belonged to engineered human single heavy domain antibodies. The remaining 15 templates were from conventional antibodies.
