# Supplementary material for: Structural diversity of B-cell receptor repertoires along the B-cell differentiation axis in humans and mice
Source: PLoS Comput Biol. 2020 Feb 18;16(2):e1007636. doi: 10.1371/journal.pcbi.1007636 (PMC7048297; doi:10.1371/journal.pcbi.1007636)
Supplement: S2 Appendix — (DOCX) [file pcbi.1007636.s018.docx]

# S2 Appendix

## Patterns of CDR-H3 cluster usage

We investigated conservatism of Structural Stem cluster usage between naïve and antigen-experienced BCR repertoires. We defined Structural Stem conservatism as the number of shared clusters between Structural Stem clusters in B-cell types (S13 Fig). In the human data, ~98% of Structural Stem CDR-H3 clusters from naïve BCR repertoires were found in antigen-experienced BCR repertoires. An analogous pattern was seen with the mouse data. Approximately 99% of Structural Stem CDR-H3 clusters in naive BCR repertoires were found in plasma IGHM BCR repertoires. Our results demonstrates that the same CDR-H3 clusters are preferentially over-represented across different B-cell types, with the number of these over-represented CDR-H3 clusters diminishing to none along the B-cell development axis. This again reinforces our findings that usage of CDR-H3 clusters becomes increasing different in BCR repertoires along the B-cell differentiation axis as only a small number of new over-represented CDR-H3 clusters are shared between antigen-experienced BCR repertoires. These over-represented clusters can be a product of antigen-specific clonally expanded B-cells.
