# Supplementary material for: Structural diversity of B-cell receptor repertoires along the B-cell differentiation axis in humans and mice
Source: PLoS Comput Biol. 2020 Feb 18;16(2):e1007636. doi: 10.1371/journal.pcbi.1007636 (PMC7048297; doi:10.1371/journal.pcbi.1007636)
Supplement: Attachment — Submitted filename: Response to reviewers. PLOS Comp Bio.docx [file pcbi.1007636.s020.docx]

Text in blue colour denotes our response to the reviewer.

Red colour indicates changes that we introduced in our manuscript.

Reviewer #1: In this study, Kovaltsuk et al. study the structural diversity of BCR repertoires along human and murine B-cell ontogeny.

Structural diversity along B-cell ontogeny has so far not been investigated. To achieve this goal, the authors improved on a previously published software for the structural annotation of antibodies (SAAB --> SAAB+) and leveraged two large-scale mouse and human BCR repertoire datasets that comprise different B-cell stages and isotypes. Applying SAAB+ to these datasets, the authors found differences in structural templates used by mice and humans and also a restriction in structural diversity across B-cell ontogeny. Previously, antibody repertoire analyses were limited to sequence-only analysis. Here, Kovaltsuk and colleagues point the way towards structural analyses on entire repertoires across adaptive immune cell thus complementing sequence-only analyses.

This is a very well executed study and i have only minor comments:

We would like to thank the reviewer for their comments which have improved the quality of this work.

- Methods:

-- It has not really become clear to this reviewer what the advancement of saab+ over saab is? Can the authors make this more explicit and dedicate a larger portion on this also to the main text.

We thank the reviewer for the comment and agree that the distinction between SAAB and SAAB+ was not accentuated enough in the manuscript. In contrast to SAAB, SAAB+ is the complete pipeline for rapid annotation of BCR repertoires with structural annotation. We have incorporated extra steps into SAAB+: filtering BCR repertoire sequences for structural viability, rapid canonical class identification, clustering of CDR-H3 structural templates and sequence/structure handling in the IMGT scheme (it is Chothia in SAAB). The output format of SAAB+ is a flat text file, which can be easily merged with outputs produced by Adaptive Immune Receptor Repertoire (AIRR) Community tools. Currently, SAAB+ takes input as a FASTA file, but it could easily work with AIRR standards, when the antibody amino acid column is introduced.

Lines 109 - 123

SAAB+ is our analysis pipeline that performs annotation of BCR repertoire amino acid data with structural information. The SAAB+ pipeline was built on the previously developed tool, SAAB [15]. The major differences/improvements of SAAB+ over SAAB are the following. First, SAAB+ scrutinises each antibody sequence for structural viability. In this step, sequences are passed through multiple structural filters (alignment to Hidden Markov Model (HMM) profiles, indel and conserved residue identification, chimeric sequence removal, presence of all 3 CDR loops according to the IMGT numbering [19] definition) [17,20]. SAAB+ utilizes SCALOP [21] for rapid canonical class identification, which gives a significant boost to the analysis rate without compromising accuracy. In a similar fashion to SAAB, FREAD is employed to predict whether CDR-H3s from the Ig-seq data share a similar structure to a crystallographically-solved CDR-H3 structure [22]. CDR-H3 predictions made by FREAD are annotated with the PDB code of the crystallographically-solved CDR-H3 structure (template). The SAAB+ pipeline also performs structural clustering of CDR-H3 templates. To find structural templates with similar CDR-H3 loop shapes (analogous to canonical loop shapes), SAAB+ structurally clusters them based on their backbone atom RMSD values (see Methods). SAAB+ outputs a tab delimited text file that can easily be merged with the current AIRR-seq standard format file [23].

-- Can the authors also indicate how much time it took to annotate the two datasets (plus hardware requirements).

We agree with the reviewer that it is important to know how long it takes to analyse a standard size BCR repertoire. SAAB+ can analyse ~5 million sequences a day on a 40 core computer cluster (Intel Xeon E5-2699 v4 @ 2.20GHz).

Lines 126 - 130

Predicting approximate shapes of CDRs rather than creating refined three-dimensional antibody models allows the SAAB+ pipeline to structurally annotate BCR repertoires rapidly. SAAB+ can analyse ~4.5 million BCR sequences a day on a 40 core computing cluster (Intel Xeon E5-2699 v4 @ 2.20GHz). SAAB+ structurally annotated the human data within two days and the mouse data in 4-5 weeks.

-- line 36: system(without s).

Thank you. Fixed now

Line 36

B-cell receptors (BCR) are the major components of the adaptive immune system.

-- for example line 42: in this work, the authors mix paratope with CDR. While the CDRs contain the paratope, the paratope is mostly one a portion of the CDR and a function of the epitope. Thus, it would be great if the authors could adjust ALL relevant portions of the text accordingly.

We agree with the reviewer that there was some confusion in terms of paratope/CDR definition. We have revisited all the definitions.

Lines 22 - 32

We have used novel rapid methods to structurally characterize the complementary-determining regions (CDRs) of more than 180 million human and mouse B-cell receptor (BCR) repertoire sequences. These structurally annotated CDRs provide unprecedented insights into both the structural predetermination and dynamics of the adaptive immune response. We show that B-cell types can be distinguished based solely on these structural properties. Antigen-unexperienced BCR repertoires use the highest number and diversity of CDR structures and these patterns of naïve repertoire CDR usage are highly conserved across subjects. In contrast, more differentiated B-cells are more personalized in terms of CDR structure usage. Our results establish the CDR structure differences in BCR repertoires and have applications for many fields including immunodiagnostics, phage display library generation, and “humanness” assessment of BCR repertoires from transgenic animals.

Lines 37 - 39.

Each individual has a huge BCR repertoire, where each individual BCR has a specific binding site composed of the complementary-determining regions (CDRs) capable of recognising a specific antigen.

Lines 41 - 50.

Here we report our novel rapid pipeline, SAAB+, which has enabled us to interrogate how the structure of the CDR changes in BCR repertoires along the B-cell differentiation axis. By analysing human and mouse BCR repertoires at an unprecedented scale, we observed species-specific structural predetermination and detected CDR dynamics across multiple stages of B-cell differentiation. We showed that naïve repertoires share the highest number and diversity of CDR structures, a pattern which was highly conserved in all B-cell donors. Our results suggest that increased B-cell differentiation is associated with a personalization of CDR structure usages. Finally, we established the differences in CDR usages between humans and mice, analysis with immediate relevance for BCR repertoire “humanness” assessment and rational immunotherapeutic engineering.

Lines 90 - 92.

These results provide crucial information about the structural changes in antibody CDRs during B-cell differentiation, with a plethora of prospective applications in immunodiagnostics and rational immunotherapeutic engineering.

Lines 206 - 208.

As B-cells differentiate to the next developmental stage, their repertoires become more personalized; a fine-tuning of antibody CDR structures along the differentiation axis.

Lines 243 - 245.

Our results again give structural confirmation of the affinity maturation process, where only CDR structures that are specific to cognate antigens are retained.

Lines 355 - 356.

This shows how structure changes as B-cells, whose CDRs are complementary to cognate antigens, are positively selected.

-- line 142: the authors state "these results confirm a structural basis for self-tolerance". Can the authors explain this further or remove this sentence from the manuscript?

We thank the reviewer for this comment. We have removed this sentence.

Lines 156 - 157

Together, these results suggest that different species may engage different epitopes on the same antigen through inherent structural biases.

-- Data availability: Has the structural annotation of the two datasets also been deposited? If not, it would be great if the authors could do so so that other groups can build off of their efforts.

We have made the data available on the OPIG website (http://opig.stats.ox.ac.uk//resources). We provided two downloadable zipped files that contain structurally annotated human and mouse BCR repertoires respectively. Each BCR repertoire contains the following information: selected CDR-H3 templates, ESS scores (template scores), Framework selected, CDR-H3 cluster, Canonical classes, and metadata.

Lines 494 - 495

Structurally annotated human and mouse BCR datasets can be downloaded from http://opig.stats.ox.ac.uk//resources webpage.

-- The authors performed subsampling experiments to account for the smaller number of PC (antigen-experienced) sequences. However, in antigen-experienced repertoires, the sequence similarity among populations will also likely be higher. Have the authors accounted for that? Or, in other terms, to what extent is the restriction in structural diversity a consequence of the increase sequence similarity?

-- the authors state that structural diversity becomes restricted with B cell ontogeny. Would this mean that the recognition potential become more restricted as well? What does this mean for the memory response which is supposed to be a trade-off between increased diversity and increased affinity?

We agree with the reviewer that sequence similarity in antigen experienced cells will be higher as it was demonstrated in original publications of the mouse data (Greiff et al.,) and the human data (Galson et al.,). Our structural analysis has shown that structural similarity is much higher than sequence similarity in BCR repertoires. In SAAB+, the increase in sequence similarity between plasma BCR sequences could lead to higher chances of picking a less diverse array of CDR-H3 templates based on the highest homology scores. Furthermore, structural annotation can also identify sequence dissimilar BCR sequences that are located in the same structural space. SAAB+ can detect both these sequence/structural paradigms, which contributes to the decrease in the structural diversity.

It is a very interesting question that is raised. We agree with the reviewer that the memory response must be diverse as a result of increased levels of somatic hypermutation. In the original publication by Galson et al., they used clonality as the measure of repertoire diversity, where a low clonality score indicates high repertoire sequence diversity. They showed that clonality was the lowest in naive B-cell repertoires and it increased along the B-cell differentiation axis in the human data. In Greiff et al., they developed a custom metric to measure repertoire diversity, the Repertoire Similarity Index (RSI). Low RSI indicates high the clonal sequence diversity. They also showed that RSI was the lowest in naive and pre B-cells, whilst plasma IgG cells had a very high value of RSI.

Both these studies researched the action of vaccination on the B-cell repertoire diversity. Hence, the repertoire sequence as well as structural diversities might be different to the resting state. We have reiterated this in the discussion.

Lines 357 - 374

The human and mouse data used in this work came from two studies that interrogated BCR repertoire sequence diversities following antigenic stimulation [7,9]. In their original publications, the sequence diversity was shown to decrease along the B-cell differentiation axis, with the most marked decline in the plasma cell repertoires in response to antigen exposure [7,9]. In our work, we find evidence to support these diversity changes on the structural level, which can be indicative of affinity selection of antigen specific BCRs. The SAAB+ pipeline was able to capture both these sequence/structural convergence paradigms, where similar CDR-H3 templates are more likely to be selected in less sequence diverse BCR repertoires as well as identification of sequence dissimilar CDR-H3 loops located in the same structural space.

Most publicly available Ig-seq studies interrogate BCR repertoires either in disease or antigenic stimulation settings [17]. This could potentially lead to sequence/structural diversity measurements that are different from the resting BCR repertoire state. Recently, two Ig-seq studies released cumulatively more than 7 billion BCR reads of unsorted B-cells from healthy individuals [2,30]. These studies provided crucial insights into the BCR sequence diversity and dynamics amongst healthy individuals. However, analogous large-scale studies have not yet been performed on sorted BCR repertoires due to high costs concomitant with experimental setups, and labour requirements. In order to develop a robust immunodiagnostics pipeline, it is important to understand sequence/structural diversity on the level of the whole repertoire as well as individual B-cell types.

- Figure aesthetics:

-- the figures are fine but not the most attractive. I don't have anything specific against them but if the authors could dedicate a few hours to making them a bit more visually pleasing, the readers would be very thankful i think.

We thank the reviewer for the comment. We have now re-plotted the figures that are found in the main manuscript text.

Figure 2:

Figure 3:

Figure 5:

**Reviewer #3**

Reviewer #3: The authors analyzed heavy chain sequences of human and mouse B-cell repertoires that were obtained from Observed Antibody Space database. They used antibody modelling tools (SCALOP and FREAD) to annotate H1, H2 and H3 loops by their putative structure/conformation. Using the structural annotation the authors showed that paratopes in naive B-cell populations are structurally distinct from paratopes in mature B-cells. Furthermore, they observed different paratope usage between human and mice.

Overall, this is a well executed work that will be appreciated by researchers involved in immunotherapy and antibody engineering.

We would like to thank the reviewer for the comments.

I would like to raise following points:

1. Due to the limitation of modelling tools the H3 loop structure predictions were carried out only for loops shorter than 16 amino acids. As a result less than 50% of observed human heavy chain H3 loops were annotated. The authors should discuss what impact (if any) may the missing annotations have on the conclusions regarding changes of paratope usage upon B-cell differentiation.

We agree with the reviewer that current modelling tools have limitations. Prediction accuracy is known to drop for longer loops due to structural freedom allowed to these loops compared to the number of solved antibody structures.

We are certain that as the availability of structural templates increases, both the coverage of BCR repertoires and homology modelling prediction accuracy will improve. For instance, in 2019 more than 400 antibody structures were solved which constitutes more than 10% of our template database. With this steadily increasing rate of antibody structure availability, further studies will be necessary to investigate tradeoffs between BCR repertoire coverage and prediction accuracy.

We agree with the reviewer that current modelling limitations leave a big portion of human BCR repertoires unexplored. We have now added a section on this in the discussion section.

Lines 386 - 399

Structural coverage of CDR-H3s of the human and mouse data was ∼48% and ∼88% respectively. In this analysis, we did not consider BCR sequences with CDR-H3 loop lengths greater than 16 amino acids to ensure high prediction accuracies. This modelling quality filter removed 3.9% (± 2 s.t.d) of the mouse and 29% (±11 s.t.d) of the human BCR datasets from the analysis, meaning that the structural diversity information of longer CDR-H3 loops unexplored (S3 Fig). Despite these marked differences in BCR repertoire coverages, both human and mouse data showed similar patterns of structural diversities along the differentiation axis. These findings agree with and provide structural reasons for the sequence diversity measurements calculated across all lengths in the original studies of the human and mouse data. Therefore, it is unlikely that the unexplored portions of human BCR repertoires would have significantly different structural diversity properties.

More than 400 antibody structures have been solved in 2019 [24], which constitutes more than 10% of our CDR-H3 template library. With this steadily increasing rate of antibody structure availability and continuous improvements in homology modelling technologies, further studies will be soon necessary to investigate tradeoffs between BCR repertoire coverage and prediction accuracies.

2. What precluded a similar analysis of light chain sequences? Could authors discuss what can we expect: similar findings or, on the contrary, learn something about distinct roles of heavy and light chains?

We thank the reviewer for this good question. We have incorporated light chain structural annotation into our SAAB+ analysis. It is deposited on github as v1.1.0 release.

We agree with the reviewer that the understanding of the light chain structural diversity is fundamentally important and there is a lot of evidence in the literature that the light chain contributes to the paratope configuration. Therefore, we would expect to see notable changes in structural diversity in light chain BCR repertoires as well.

The main reason why the light chain analysis was not included is the scarcity of sorted light chain BCR repertoires from different species in the public domain.

We have now discussed the importance of the light chain information in the manuscript.

Lines 375 - 385

In this work we considered only the three CDRs encoded by heavy chain genes. However, the light chain also plays an important role in shaping the BCR paratope [31]. Therefore, it is anticipated that the diversity of light chain CDR configurations would also change along the B-cell differentiation axis. To advance our understanding of the role of the light chain in paratope shaping, further investigations are required to study joined structural diversities of heavy and light chains within individual B-cell donors.

Inclusion of cognate light chain pairing information into our analysis would also facilitate generation of refined antibody models. Increased availability of paired heavy/light BCR data [32] and improvements in antibody modelling speed [13] will pave the way to a new frontier of antibody structure usage analysis at the scale of an entire BCR repertoire. Structural descriptors harvested from these models will push forward the resolution of our current work, enabling calculations of paratope charge and hydrophobicity, as well as antibody developability profiles [33].
